# Multi-Antenna GNSS–Accelerometer Fusion Attitude Correction Algorithm for Offshore Floating Platform Displacement Monitoring

**DOI:** 10.3390/s24237804

**Published:** 2024-12-06

**Authors:** Xingguo Gao, Junyi Jiang, Guoyu Xu, Zengliang Chang, Jichao Yang

**Affiliations:** 1 Shandong Electric Power Engineering Consulting Institute Co., Ltd., Jinan 250013, China; gaoxingguo@sdepci.com (X.G.); jiangjunyi@sdepci.com (J.J.); changzengliang@sdepci.com (Z.C.); 2 College of Ocean Science and Engineering, Shandong University of Science and Technology, Qingdao 266590, China; 202483190061@sdust.edu.cn

**Keywords:** multi-antenna GNSS, GNSS and accelerometer fusion, displacement and attitude monitoring system, offshore floating platform

## Abstract

In order to solve the problem of fixed ambiguity and decreased accuracy in GNSS displacement monitoring of the offshore floating platforms, an attitude correction algorithm based on the fusion of a multi-antenna GNSS and an accelerometer was proposed using the Kalman filtering method. The algorithm was validated on a physical simulation platform and a real offshore floating platform. The results indicate that this fusion method effectively compensates for the loss of high-frequency displacement information caused by low GNSS sampling rates, improves situations in which the fusion effect deteriorates due to attitude changes, and enhances the accuracy of GNSS and accelerometer fusion monitoring through offshore buoy testing.

## 1. Introduction

Floating offshore structures have been extensively utilized in marine civil engineering construction, as well as offshore and port operations [1]. Unlike onshore buildings, offshore platforms are subject to various factors such as waves, weather conditions, tidal currents, and other elements during their operation. The challenging working environment of offshore platforms results in displacement, tilt, rotation, and other risks, which ultimately compromise their stability and have a detrimental impact on long-term production safety [2]. Currently, during the operation process, offshore platforms primarily depend on regular maintenance and flaw detection to evaluate the structural safety of the platform [3]. However, the real-time capabilities are limited, and it is not possible to obtain a more comprehensive assessment of structural deformation, overall displacement, and other related information. Attitude monitoring can aid in the timely detection and monitoring of these changes, providing real-time updates on the operational status of the platform. Therefore, in order to enhance the monitoring and evaluation of the marine environment’s influence on offshore floating platforms, it is imperative to implement deformation monitoring technology in offshore settings and establish a comprehensive set of monitoring systems that are specifically designed for offshore floating platforms.

The Global Navigation Satellite System (GNSS) is characterized by its lack of visibility requirements, ease of deployment, ability to operate in all weather conditions, and automation capabilities [4]. It has been extensively utilized in the domain of dynamic deformation monitoring for tall buildings [5], bridges [6], landslides [7], and other related applications. The challenge in maritime applications remains the issue of reducing the rate of ambiguity fixation and improving accuracy, which is caused by the increase in baseline length [8]. By acquiring the position information of multiple antennas, it is possible to quickly and accurately obtain attitude information [9,10,11]. GNSS has significant potential for application in attitude measurement, not only as an independent attitude measurement system but also to assist in the quick initialization and calibration of, for instance, inertial navigation systems [12]. In recent years, numerous scholars have also conducted studies on the utilization of GNSS for maritime attitude measurements and have obtained favorable outcomes [13,14,15,16]. Accelerometers have the capability to provide real-time high-frequency acceleration data. Accelerometers can effectively detect high-frequency vibrations, addressing the limitations of low sampling rates in GNSS. This enables accurate identification of high-frequency vibrations in the target object. Additionally, accelerometers complement GNSS measurements effectively [17]. The current field of data fusion for GNSS and accelerometers primarily focuses on enhancing Kalman filtering. Various methods have been proposed for the data fusion stage, including multi-rate Kalman filtering [18], multi-rate anti-differential Kalman filtering [19], and adaptive multi-rate Kalman filtering [20]. Processing tools, such as forward filtering [21], forward filtering backward smoothing [22], and the RTS fixed-interval optimal smoothing algorithm [23], have also been proposed for the result processing stage. This fusion technology allows for continuous improvement and application in a broader range of scenarios, while also providing more precise and dependable localization results. However, the majority of the aforementioned studies focus on land-based environments, with only a limited number of studies examining maritime application scenarios. When a carrier is in motion on the surface of the sea, its orientation constantly changes due to the waves. As a result, the accelerometer measures triaxial acceleration that cannot be directly combined with GNSS data to determine displacement.

The application of GNSS monitoring technology, along with the fusion of GNSS and accelerometers, has significant research and application value in terms of accurately extracting the displacement and attitude change information of offshore floating platforms. However, this approach also presents challenges. The utilization of GNSS monitoring technology in marine environments serves two main purposes. Firstly, it addresses the challenge of maintaining accurate GNSS positioning and posturing as the baseline length increases. Secondly, it tackles the issue of achieving data fusion between GNSS and accelerometers in order to obtain high-frequency and high-precision displacement information when the offshore platform’s attitude changes. For the fusion of GNSS and accelerometers, this paper proposes utilizing the attitude obtained from the multi-antenna GNSS to correct the accelerometer. The fusion of GNSS and accelerometer displacement data in scenarios involving an attitude change is achieved. Finally, a simulation experiment illustrates the importance of accelerometer attitude correction and the enhancement of the fusion effect by attitude correction.

## 2. Materials and Methods

### 2.1. Principle of Accelerometer Displacement Measurement

An accelerometer is capable of providing acceleration information in the axial direction of installation. By integrating the acceleration, velocity information and displacement information can be obtained. There are two commonly used methods for obtaining displacement through accelerometer integration: time-domain integration and frequency-domain integration.

#### 2.1.1. Time-Domain Integral

The error in displacement obtained by the accelerometer through time-domain integration increases over time. Therefore, it is only suitable for measuring displacement over a short period. The method based on time-domain integration can be expressed as follows:

(1) $$\left\{\begin{array}{c} v\left(t\right)=v_{0}+\int_{0}^{t}a\left(t\right)dt\\ s\left(t\right)=s_{0}+\int_{0}^{t}v\left(t\right)dt \end{array}\right.$$

where $v_{0}$ and $s_{0}$ represent the velocity and displacement at the initial moment and $v\left(t\right)$ and $s\left(t\right)$ represent the velocity and displacement at moment *t*.

Since the acceleration acquired by the accelerometer inevitably contains noise, assuming that the noise is η, the true value of the acceleration at moment *t* is $\mu \left(a\right)$. The relationship between the measured value and the true value can be expressed as:

(2) $$a\left(t\right)=\mu \left(t\right)+\eta$$

Then, there is:

(3) $$v\left(t\right)=v_{0}+\int_{0}^{t}a\left(t\right)dt=v_{0}+\int_{0}^{t}\mu \left(t\right)dt+\eta t$$

(4) $$\begin{array}{ll} s\left(t\right) & =s_{0}+\int_{0}^{t}v\left(t\right)dt=s_{0}+\int_{0}^{t}\left(v_{0}+\int_{0}^{t}\mu \left(t\right)dt+\eta t\right)\mu \left(t\right)\\ & =s_{0}+v_{0}t+\frac{1}{2}\eta t^{2}+\int_{0}^{t}\int_{0}^{t^{\prime }}\mu \left(t\right)dt^{\prime }dt \end{array}$$

From Equations (3) and (4), the cumulative error after one integration of the acceleration is ηt. The cumulative error after quadratic integration of the acceleration is $\eta t^{2}/2$. To attenuate the effect of noise, the raw acceleration data usually need to be filtered and the trend term removed before the quadratic integration of the acceleration.

For discrete acceleration data, the commonly used integration methods are the trapezoidal integration method and the Simpson integration method. The trapezoidal integration method is expressed as:

(5) $$\left\{\begin{array}{c} v\left(i\right)=v\left(i-1\right)+\frac{a\left(i-1\right)+a\left(i\right)}{2}\cdot \Delta t\\ s\left(i\right)=s\left(i-1\right)+\frac{v\left(i-1\right)+v\left(i\right)}{2}\cdot \Delta t \end{array}\right.$$

Simpson’s integral method is expressed as:

(6) $$\left\{\begin{array}{c} v\left(i\right)=v\left(i-1\right)+\frac{a\left(i-1\right)+4a\left(i\right)+a\left(i+1\right)}{6}\cdot \Delta t\\ s\left(i\right)=s\left(i-1\right)+\frac{v\left(i-1\right)+4v\left(i\right)+v\left(i+1\right)}{6}\cdot \Delta t \end{array}\right.$$

where i=1,2,⋯N, Δt is the sampling interval.

#### 2.1.2. Frequency-Domain Integral

The displacements obtained through frequency-domain integration are not subject to error accumulation, unlike time-domain integration, as high-frequency and low-frequency noise components are eliminated. However, the frequency-domain integral can only ensure the reliability of the relative accuracy as the initial position and velocity are not determined.

The continuous acceleration data are represented as $a\left(t\right)$. The actual output from the accelerometer is a discrete sequence of accelerations at Δt sample intervals, denoted by $a\left(n\right)$, $n=0,1,2,\cdots \left(N-1\right)$. The discrete Fourier transform of a discrete sequence $a\left(n\right)$ can be expressed as:

(7) Ak=DFTan=∑n=0N−1ane−j2πnkN k=0,1,2⋯,N−1

The inverse transform of the discrete Fourier transform can be expressed as:

(8) an=1N∑k=0N−1Ake−j2πnkN n=0,1,2⋯,N−1

By the integral nature of the Fourier transform, the discrete Fourier transform of $v\left(n\right)$ and $s\left(n\right)$ can be expressed as:

(9) $$V\left(k\right)=\frac{1}{jw\left(k\right)}A\left(k\right)=\frac{N}{j2\pi k}A\left(k\right)\;k=0,1,2\cdots ,\left(N-1\right)$$

(10) $$S\left(k\right)=\frac{1}{{\left(jw\left(k\right)\right)}^{2}}A\left(k\right)=-\frac{N^{2}}{{\left(2\pi k\right)}^{2}}A\left(k\right)\;k=0,1,2\cdots ,\left(N-1\right)$$

After obtaining the Fourier transforms $V\left(k\right)$ and $S\left(k\right)$ of the velocity and displacement through Equations (9) and (10), the inversion operation is performed to obtain the velocity and displacement sequences $v\left(n\right)$ and $s\left(n\right)$, denoted as:

(11) vn=1N∑n=0N−1Vke−j2πnkN=∑n=0N−11j2πkAke−j2πnkN n=0,1,2⋯,N−1

(12) sn=1N∑n=0N−1Ske−j2πnkN=−∑n=0N−1N2πk2Ake−j2πnkN n=0,1,2⋯,N−1

From Equations (11) and (12), it can be seen that the integration in the frequency domain can well avoid the cumulative amplification of the error brought by the integration in the time domain. The scale factor $1/\left(j2\pi k\right)$ and $-1/{\left(j2\pi k\right)}^{2}$ contained in the velocity and displacement during discrete Fourier transform represent that a decrease in velocity and displacement at 3 dB and 6 dB per octave, respectively [24].

### 2.2. Fusion of GNSS and Accelerometer Based on Kalman Filter

To achieve real-time fusion of GNSS and accelerometer data, the displacement sequence obtained from GNSS and the displacement sequence obtained from integrating the accelerometer data are commonly fused using Kalman filtering. This section provides a brief description of the principles of Kalman filtering used for fusion, as well as the model for fusing GNSS and accelerometer data.

#### 2.2.1. Kalman Filter Fundamentals

Kalman filtering was developed by Swerling [25] and Kalman [26] as a prediction and filtering technique for linear Gaussian systems. Since its introduction, the Kalman filter has been extensively applied in various fields and is particularly prevalent in navigation [27,28,29]. The Kalman filtering method is considered a classical approach and will not be further elaborated upon in this paper.

#### 2.2.2. Kalman Filter Fusion of GNSS and Accelerometers

Assuming that the three axes of the accelerometer are mutually perpendicular, with the x-axis oriented towards the east, the y-axis oriented towards the north, and the z-axis oriented towards the sky, and assuming that the acceleration remains constant (according to the constant acceleration model) during one sampling period and its variation follows a Gaussian white noise distribution with a mean value of zero, the Kalman filter state equation for the fusion of GNSS and the accelerometer can be expressed as:

(13) $$\left[\begin{array}{c} d\left(t\right)\\ v\left(t\right) \end{array}\right]=\left[\begin{array}{cc} 0 & 1\\ 0 & 0 \end{array}\right]\left[\begin{array}{c} d\left(t\right)\\ v\left(t\right) \end{array}\right]+\left[\begin{array}{c} 0\\ 1 \end{array}\right]a\left(t\right)+\left[\begin{array}{c} 0\\ 1 \end{array}\right]w\left(t\right)$$

where $d\left(t\right)$ denotes the accelerometer’s integral displacement, $v\left(t\right)$ denotes the accelerometer’s integration velocity, $a\left(t\right)$ denotes the acceleration, and $w\left(t\right)$ denotes the system noise.

The observation equation can be expressed as:

(14) $$y\left(t\right)=\left[\begin{array}{cc} 1 & 0 \end{array}\right]\left[\begin{array}{c} d\left(t\right)\\ v\left(t\right) \end{array}\right]+v\left(t\right)$$

where $y\left(t\right)$ denotes the GNSS displacement.

Constructing the state and measurement equations according to the principle of Kalman filtering can be expressed as:

(15) $${\hat{x}}_{m}=\left[\begin{array}{c} d\\ v \end{array}\right],\;\Phi_{m}=\left[\begin{array}{cc} 1 & \Delta t\\ 0 & 1 \end{array}\right],\;G_{m}=\left[\begin{array}{c} \frac{\Delta t^{2}}{2}\\ \Delta t \end{array}\right],\;u_{m}=a_{m}$$

The state noise covariance Q and the observation noise covariance R can be expressed as:

(16) $$Q=q\left[\begin{array}{cc} \Delta t^{3}/3 & \Delta t^{2}/2\\ \Delta t^{2}/2 & \Delta t \end{array}\right]$$

(17) $$R=\frac{r}{\Delta t}$$

where Δt denotes the sampling interval, q denotes the variance of the accelerometer, and r denotes the displacement variance of the GNSS.

The Kalman-filtered equation of state for the fusion of GNSS and the accelerometer can be expressed as follows when the three axial displacements are considered simultaneously in the navigation coordinate system:

(18) $$X_{m}=\left[\begin{array}{cc} I_{3\times 3} & \Delta t\cdot I_{3\times 3}\\ 0_{3\times 3} & I_{3\times 3} \end{array}\right]X_{m-1}+\left[\begin{array}{c} 0.5\cdot \Delta t^{2}\cdot I_{3\times 3}\\ \Delta t\cdot I_{3\times 3} \end{array}\right]a_{m-1}+Q_{m-1}$$

where $X_{m}={\left[\begin{array}{cccccc} d_{x} & d_{y} & d_{z} & v_{x} & v_{y} & v_{z} \end{array}\right]}^{T}$ indicates the displacement and velocity of the three axes.

The observation equation can be expressed as:

(19) $$L_{m}=\left[\begin{array}{cc} I_{3\times 3} & 0_{3\times 3} \end{array}\right]X_{m}+R_{m}$$

where $L_{m}=\left[\begin{array}{ccc} x_{gnss} & y_{gnss} & z_{gnss} \end{array}\right]$ denotes the displacement of the GNSS.

### 2.3. Acceleration and GNSS Displacement Fusion Based on Attitude Correction

In Section 2.2.1, it is explicitly assumed that the axes of the acquired acceleration align with the navigation coordinate system. In situations where the attitude remains constant, fixed parameters can be employed for fusing the GNSS and accelerometers. However, in scenarios where the attitude may vary, it is essential to consistently consider the actual attitude of the accelerometers.

**Figure 1:**
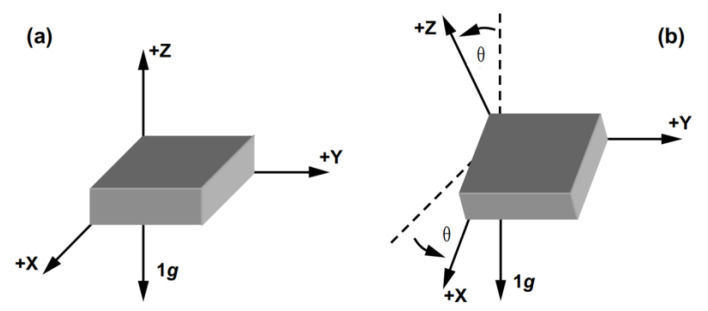
Accelerometer tilt diagram. (**a**): schematic diagram of coordinate axis pointing; (**b**): schematic diagram of coordinate axis pointing changes under posture changes.

Assuming that the three axes of the accelerometer and the navigational coordinate system (ENU) align perfectly under ideal conditions (Figure 1a), the acceleration caused by the movement of the carrier at this specific location can be mathematically represented as:

(20) $$a_{n}={\left[\begin{array}{ccc} a_{e} & a_{n} & a_{u} \end{array}\right]}^{T}$$

where $a_{n}$ denotes the acceleration in the navigation coordinate system and $a_{e}$, $a_{n}$, and $a_{u}$ represent the triaxial acceleration due to carrier motion, respectively.

The acceleration of gravity on the carrier can be expressed as:

(21) $$g_{n}=\left[\begin{array}{ccc} 0 & 0 & -g \end{array}\right]$$

where g denotes the local gravitational acceleration in a vertical downward direction.

Since the axial direction of the accelerometer aligns with the navigation coordinate system, the acceleration output from the accelerometer can be represented as:

(22) $$a_{b}=a_{n}+g_{n}$$

At this point, only the acceleration due to gravity needs to be subtracted to obtain the acceleration due to the motion of the carrier.

Taking the x-axis of accelerometer as an example, when the axial direction is tilted (Figure 1b), the acceleration measured by the accelerometer along the x-axis is no longer solely the acceleration of the navigational coordinate system. Instead, it represents the upward projection on the x-axis of the combined force of the acceleration of the navigational coordinate system and the acceleration of gravity, i.e.,

(23) $$a_{x}=a_{e}\cos\theta +g\sin\theta$$

where $a_{x}$ denotes the measured value of the actual output of acceleration, $a_{e}$ denotes the eastward acceleration in the navigational coordinate system, and θ denotes the angle of inclination.

Then, at this point, the acceleration $a_{e}$ in the eastward direction under the navigational system should be expressed as:

(24) $$a_{e}=\frac{a_{x}-g\sin\theta }{\cos\theta }$$

In practice, when considering that all three axial directions change, the acceleration obtained from the acceleration triaxial measurements can be expressed as:

(25) $$a_{b}=C_{n}^{b}a_{n}+C_{n}^{b}g$$

where $C_{b}^{n}$ denotes the attitude transfer matrix of the accelerometer relative to the navigation coordate system.

If the actual attitude of the accelerometer and the local gravitational acceleration are known, the three-axis acceleration due to the motion of the carrier in the navigation coordinate system can be expressed as:

(26) $$a_{n}=C_{b}^{n}\left(a_{b}-C_{n}^{b}g\right)$$

By employing the multi-antenna GNSS technique, it is possible to acquire real-time attitude data for the carrier and to convert the acceleration output from the accelerometer into acceleration in the navigation coordinate system. By utilizing the corrected acceleration and by referring to the fusion method outlined in Section 2.2.2, it is feasible to achieve GNSS and accelerometer fusion based on attitude correction.

### 2.4. Experiment Introduction

#### 2.4.1. Vibration Experiment on a Physical Simulation Platform

**Figure 2:**
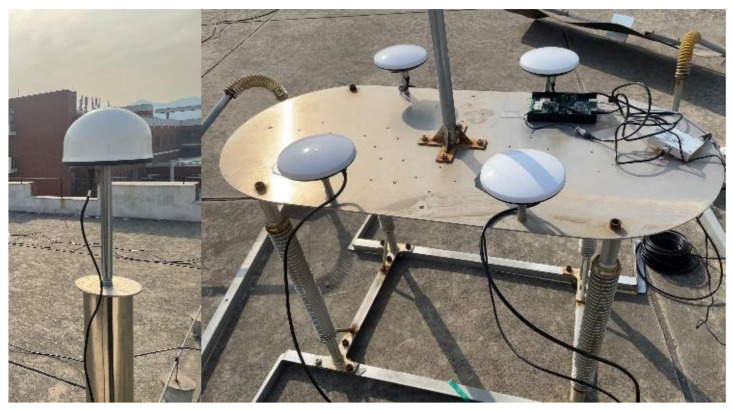
Base station and simulation platform.

**Figure 3:**
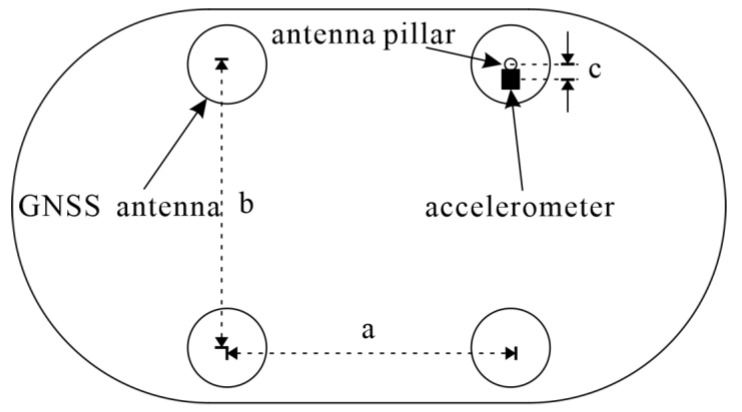
Installation diagram of GNSS antenna and accelerometer. a: the distance between the two antennas is 52.68 cm; b: the distance between the two antennas is 43.50 cm; c: the distance between the accelerometer and antenna is 2.79 cm.

In order to validate the actual impact of the algorithm, a co-vibration physical platform (Figure 2) was constructed on the rooftop of the building. The platform is 127 cm long, 60 cm wide, and 80 cm high, and is supported by four springs and equipped with four GNSS antennas and a three-axis accelerometer (Figure 2). The four GNSS antennas are distributed in a square shape with a 52.68 cm length and a 43.50 cm width (Figure 3). The accelerometer is installed near the antenna pole which located in the upper right corner (Figure 3). The correction value of the accelerometer and antenna phase center plane is (2.79 cm, 0, −6.15 cm). In addition, a GNSS base station was installed on the rooftop (Figure 2). The GNSS data collection uses a UB482 receiver (Unicore, Shenzhen, China), the specific parameters of which are shown in Table 1. The performance parameters of the accelerometer are shown in Table 2.

A hardware timer was designed for time synchronization: the counter in the timer resets to zero after each PPS signal arrives; whenever the accelerometer signal arrives, the counter value is read to calculate the arrival time of each accelerometer signal in that second. By adding the current UTC time, the final arrival time of the accelerometer signal can be obtained. Finally, this is converted to GPS TIME.

Displacement motion and attitude changes are simulated by artificially shaking the platform. A specific period of simulated vibration data is then extracted for detailed analysis to confirm the effectiveness of the fusion between the multi-antenna GNSS and the accelerometer.

#### 2.4.2. Multi-Antenna GNSS Buoy Experiments

**Figure 4:**
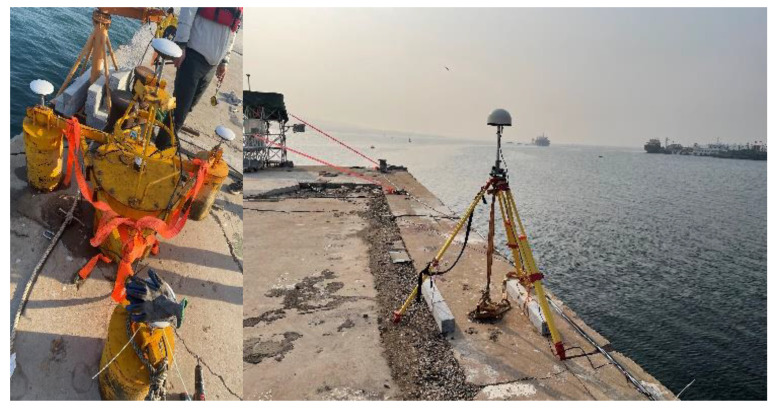
Multi-antenna GNSS buoy and GNSS reference station [30].

The area where the buoy was placed is located in Huangdao District, Qingdao City, specifically at Jimiya dock. A reference station was established at the dock, equipped with a choke antenna. The buoy itself was equipped with four Huaxin HX-CSX601A (HI TARGET, Guangzhou, China) blocking antennas, of which the one located in the center of the buoy was defined as the main antenna (Figure 4). The collection card chosen for this setup is the Hexin Star-Tone UB482 card, which is capable of receiving dual-frequency signals from all four systems. The maximum sampling rate of the buoy is 20 Hz. The buoy is powered by batteries and can maintain stable operation for more than 72 h at a time. The accelerometer used in this setup is the same as the one used in Section 2.4.1.

To validate the accuracy of multi-antenna buoy altimetry, both tide gauge and radar altimetry equipment were deployed simultaneously at the pier. This allowed for the provision of elevation information every minute. The accuracy can then be verified and compared.

## 3. Results and Discussion

### 3.1. Horizontal Displacement Experiment

**Figure 5:**
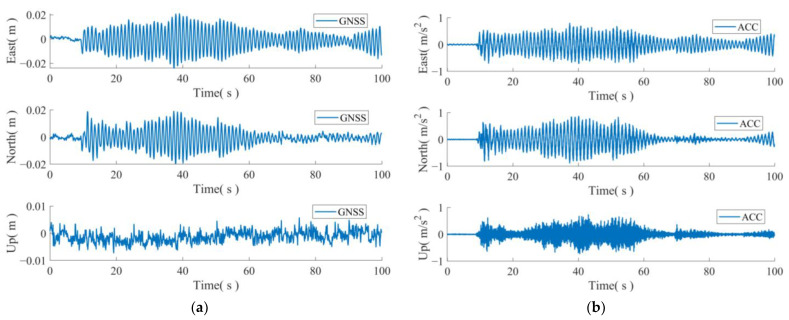
GNSS displacement sequence and triaxial acceleration sequence after removing trend term. (**a**) GNSS displacement sequences; (**b**) acceleration sequence.

In this set of experiments, only horizontal motion was utilized to simulate the scenario of a stationary platform that experiences displacement without any change in attitude. Figure 5 presents a data sample obtained from this simulation. Figure 5a displays the time series of the displacement for the three axes of the GNSS, east–north–sky, at a frequency of 10 Hz, while Figure 5b illustrates the corresponding acceleration series for the three axes at a frequency of 100 Hz. The GNSS data were acquired through RTK solving, and the left figure provides a more accurate representation of the displacement changes caused by the simulated shaking due to the high sampling rate of the GNSS data acquisition in the experiment and the proximity of the base station.

**Figure 6:**
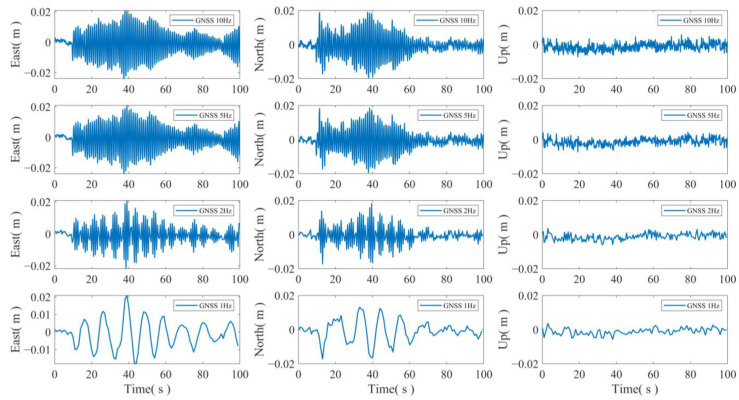
GNSS displacement sequences at different frequencies.

To ascertain the optimal sampling frequency for horizontal displacement monitoring, GNSS displacement sequences were acquired at different sampling rates of 10 Hz, 5 Hz, 2 Hz, and 1 Hz. The obtained results are presented in Figure 6.

**Figure 7:**
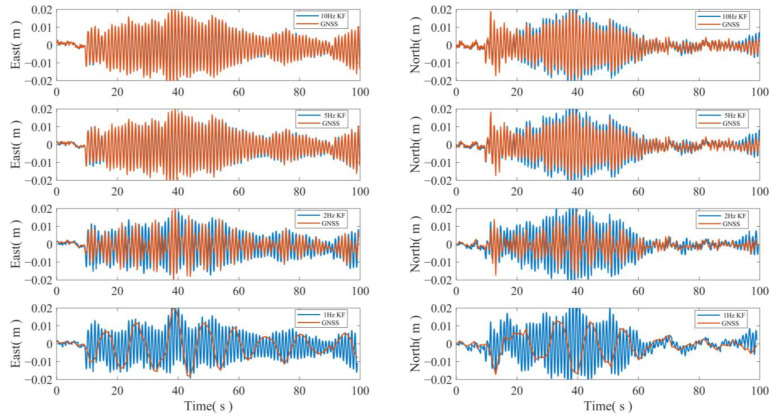
GNSS displacement and accelerometer data fusion at different frequency.

To determine the optimal sampling frequency for the combined GNSS/INS approach, a multi-rate Kalman filter fusion was conducted using THE 100 Hz acceleration data and GNSS displacement sequences at frequencies of 10 Hz, 5 Hz, 2 Hz, and 1 Hz, respectively. The results of the displacement sequences are presented in Figure 7. The figure demonstrates that the horizontal displacement sequence of the carrier can be effectively restored by fusing the GNSS displacement sequence with the accelerometer at different frequencies. Furthermore, even when the GNSS data at 1 Hz was fused with the accelerometer, the high-frequency motion of the carrier could still be accurately restored.

**Figure 8:**
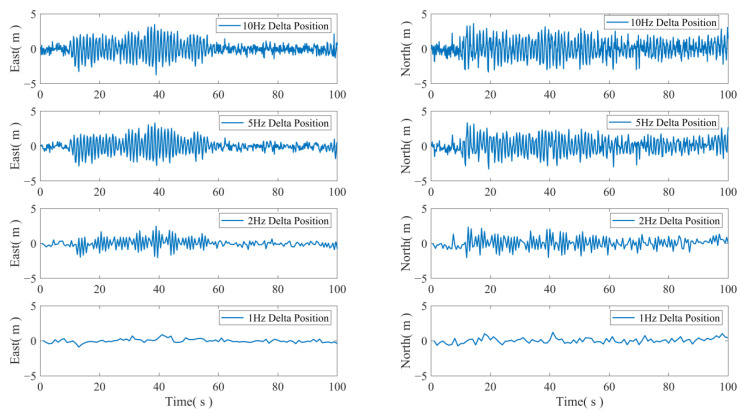
The difference between the fusion result and the GNSS result.

The displacement sequences of the four sets of fusion data between GNSS and acceleration mentioned above differ from the GNSS displacement sequences. The results obtained are shown in Figure 8. It can be observed from Figure 8 that the accelerometer predictions through filtering are closer to the GNSS measurements, but the residual values exhibit a strong regular pattern during periodic motion.

### 3.2. Attitude Change Experiment

**Figure 9:**
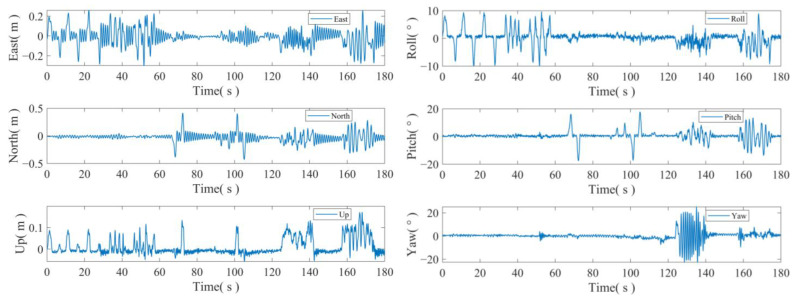
The displacement and attitude changes of the simulated platform.

In this series of experiments, the researchers incorporated attitude changes in addition to swaying to simulate the displacement and attitude changes that can occur in a real maritime environment. Figure 9 illustrates the simulated platform displacements and intercepted attitude changes over a specific time period. The left figure displays the sequence of east–north–sky displacements obtained through the use of GNSS, while the right figure shows the attitude obtained using four antennas.

**Figure 10:**
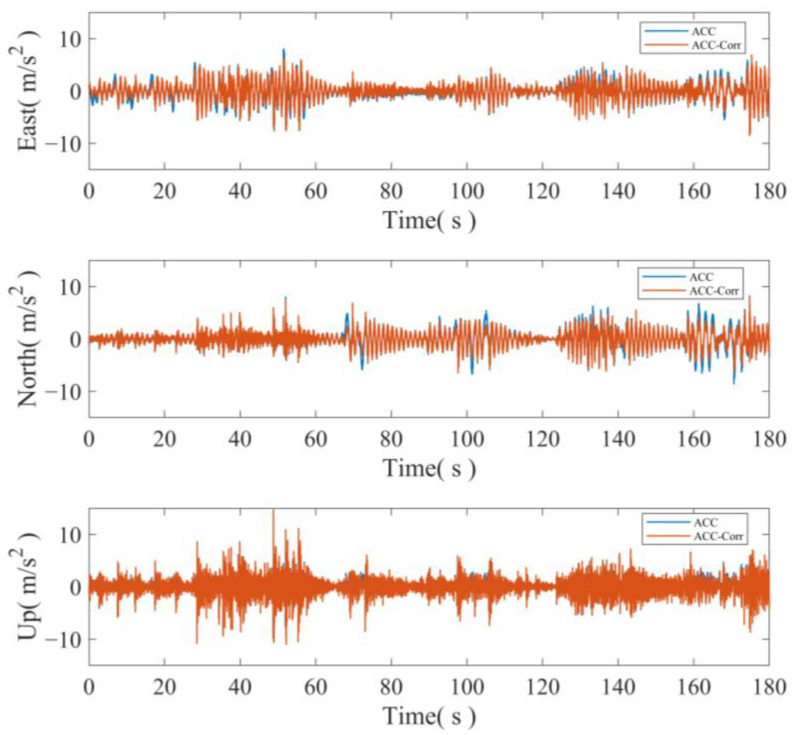
Accelerometer values before and after attitude correction.

The accelerometer is calibrated using the attitude information obtained from the multiple antennas. The three-axis acceleration before and after calibration is illustrated in Figure 10. Upon comparing the acceleration values before and after calibration, it is evident that significant errors occur in the accelerometer measurements when there are drastic changes in attitude. Therefore, calibration is necessary to achieve accurate fusion.

**Figure 11:**
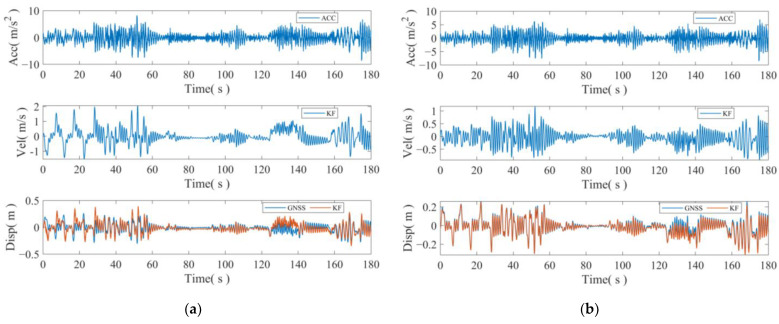

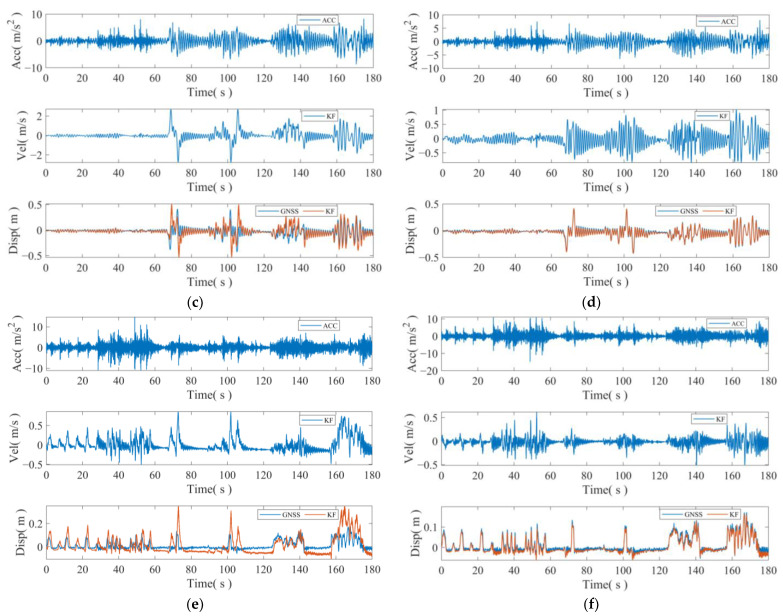
Comparison of fusion results before and after attitude correction. (**a**) Eastward acceleration before correction; (**b**) comparison of fusion effects after correction; (**c**) northward acceleration before correction; (**d**) comparison of fusion effects after correction; (**e**) skyward acceleration before correction; (**f**) comparison of fusion effect after correction. ACC: acceleration; KF: Kalman filtering results; GNSS: GNSS raw displacement sequence.

To assess the true impact of attitude correction on the accelerometer, the accelerometer data before and after correction, along with the GNSS displacement sequence, are separately utilized for fusion. The fusion results before and after correction in the three axes are illustrated in Figure 11.

Figure 11 illustrates the acceleration, Kalman-filtered fused velocities, and displacements in each axial direction, along with the original GNSS displacement sequence. The comparison in the figure reveals that utilizing the original acceleration measurements directly results in a significant error in the fusion outcome during the time period when the attitude changes. However, after incorporating the attitude correction, although some error remains, the fusion effect is considerably enhanced, and the actual displacement information is essentially restored.

### 3.3. Multi-Antenna GNSS Buoy Displacement Attitude Monitoring Applications

**Figure 12:**
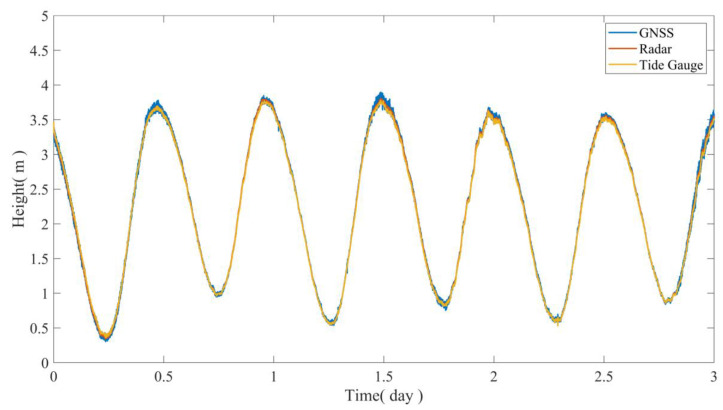
The main antenna elevation sequence of the multi-antenna GNSS, radar, and tide gauge.

The data obtained from the multi-antenna buoy were initially processed and analyzed using the algorithm proposed in this paper. Following the data processing of the buoy data from 9 February 2023, at 8:00 a.m., to 12 February 2023, at 8:00 a.m., which spans a total of three days, the resulting of GNSS main antenna elevation and attitude changes are depicted in Figure 12. 

**Figure 13:**
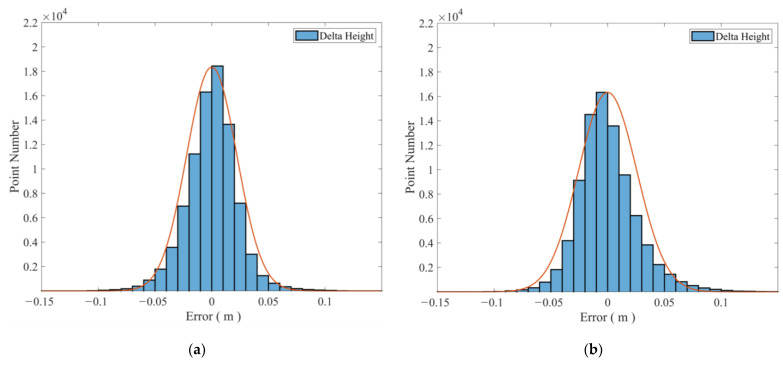
Error statistics of GNSS with radar and tide gauge data. (**a**) GNSS and radar data; (**b**) GNSS and tide gauge data.

Due to the different starting benchmarks of GNSS, tide gauges, and radar altimetry equipment, elevation correction was performed using the measured calibration values in order to compare the three sets of data. After elevation correction, three methods of measuring tidal level elevation can be obtained (Figure 13). Furthermore, the difference values between the GNSS, radar, and pressure gauge were statistically analyzed. The RMSE between the GNSS and radar was 2.218 cm, with a 95% confidence interval of ±3.665 cm (Figure 13a), while the RMSE between the GNSS and tide gauge was 2.571 cm, with a 95% confidence interval of ±4.584 cm (Figure 13b). Overall, the results obtained from the three tidal testing methods reflect consistent trends in tidal level changes, and the numerical consistency is also high.

**Figure 14:**
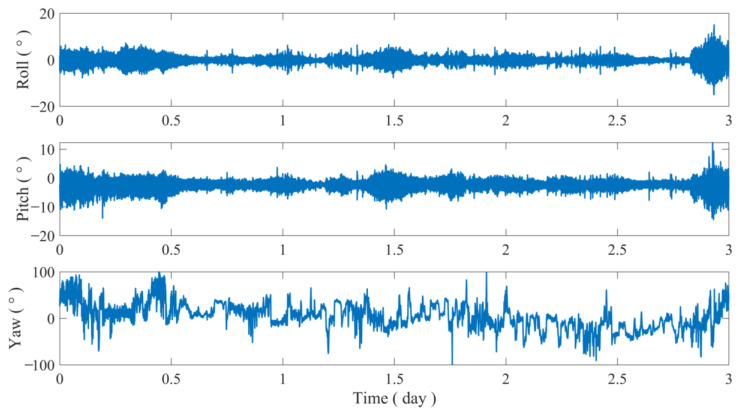
Multi-antenna GNSS buoy attitude sequence.

The attitude of the multi-antenna buoy was subsequently analyzed as a time series. The three-axis attitude series demonstrates a strong correlation between the attitude changes in the buoys and the ebb and flow of tides (Figure 14). The fluctuation in attitude increases proportionally during the range where the tide level rapidly increases or decreases. Additionally, the pitch and roll angles experience an increase during certain periods of significant elevation changes.

**Figure 15:**
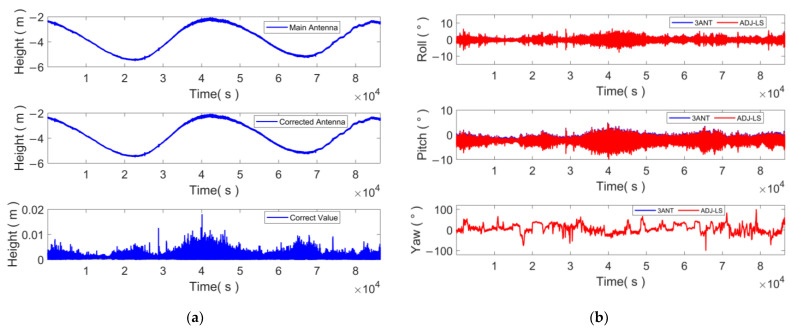
Buoy elevation and attitude sequence on day 41. (**a**): the elevation of the main antenna, the elevation after the four-antenna network leveling and attitude correction, and the elevation correction value obtained through the attitude, respectively; (**b**) the information of the buoy’s roll angle, pitch angle, and heading angle.

The GNSS elevation and attitude sequences for a specific day were utilized to conduct a detailed analysis of the data. The changes in elevation and attitude of the antenna on day 041 of the cumulative yearly days were selected for examination (Figure 15). The buoy elevation data provides information on the initial elevation of the main antenna, the elevation of the main antenna after attitude correction, and the value of the attitude elevation correction. It should be noted that the actual measured distance from the draft point of the buoy to the main antenna is 0.97 m. The attitude data provide information on the attitude measured through the direct three-antenna method, as well as the attitude computed using the multi-antenna attitude correction algorithm discussed in this paper. The findings indicate that this method enhances the accuracy of altimetry measurements by reducing errors caused by buoy fluctuations during high tide.

## 4. Discussion

### 4.1. Determination of Sampling Frequency for Horizontal Displacement Monitoring

In practical engineering applications, it is common to process multiple measurement stations. Factors such as computing power, data volume, and cost are taken into consideration when selecting the frequency of GNSS monitoring data. Typically, low-frequency GNSS monitoring data are used due to these factors. However, according to the Nyquist Sampling Theorem, the sampling frequency should be at least twice the highest frequency of the effective signal in order to accurately capture all the information of the original signal. Therefore, high-frequency GNSS monitoring data are required. To resolve this contradiction, a solution is to combine GNSS with INS. This combination allows for a highly accurate representation of platform displacement in highly dynamic scenes, with a GNSS monitoring frequency of 1 Hz.

However, in extreme weather conditions, the vibration frequency of waves can reach 10 Hz [31]. In response to this situation, it is necessary to monitor the vibration frequency in real time and establish a program that automatically switches to the 5 Hz data mode once a continuous vibration signal exceeding 1 Hz is detected, to ensure that the attitude monitoring system can more accurately reflect the high-frequency vibration of the vehicle in adverse weather conditions. This program can accurately monitor waves within 2 Hz. If the waves exceed 2Hz, it is necessary to replace the high-precision receiver.

The residual analysis in this experiment revealed a significant regularity. There are two reasons for this phenomenon. Firstly, the simulation platform is a scaled down model supported by shock-absorbing springs, and the accelerometer is fixed to one of the antenna columns. The weight of the accelerometer inevitably causes a slight system error in the posture of the entire platform during the experiment. Secondly, the GNSS antenna phase center differs from the center of the accelerometer. These two factors combined lead to a discrepancy between the actual measurement of the accelerometer and the actual carrier acceleration.

### 4.2. GNSS and Accelerometer Fusion Effect

After correcting the attitude through the fusion of the GNSS and accelerometer, there still remains some error. One contributing factor is the disparity between the phase center of the GNSS antenna and the mounting position of the accelerometer, leading to a greater swing in the GNSS compared to the accelerometer. Additionally, there is an error in the attitude measured by the GNSS and the accelerometer due to mounting inaccuracies. However, overall, the proposed attitude correction algorithm in this paper enables the fusion of GNSS and accelerometers in attitude change scenarios, resulting in an improvement to some extent.

### 4.3. Multi-Antenna GNSS Floating Platform Monitoring Validation

Practical applications have demonstrated that the attitude fluctuation of the floating platform is more obvious during the rapid rise or fall of tide level, while pitch and roll angle changes are evident during periods of significant elevation changes. Based on the actual sea conditions, this phenomenon reflects the impact of waves on the buoy, resulting in an increase in the elevation fluctuation of the buoy during these time periods. Waves are a significant factor contributing to the attitude error of floating platforms at sea. In this experiment, the antenna, positioned 0.97 m above the water surface, experiences a 2 cm error in the U direction due to wave-induced attitude changes. However, the attitude correction fusion algorithm employed in this study is capable of rectifying the error by approximately 1 cm.

## 5. Conclusions

A method was proposed for monitoring the displacement of low-speed dynamic loads at sea using a combination of a four-antenna GNSS and an accelerometer. Changes in the attitude of offshore carriers can cause axial changes in an accelerometer. Using four-antenna attitude information to correct the three-axis acceleration of the accelerometer in real time can improve the accuracy of positioning and attitude measurements of floating carriers on the sea. A Kalman filtering model integrating GNSS and an accelerometer was designed, and the algorithm was validated using a simulation platform. The experimental results show that the proposed accelerometer attitude correction method can effectively compensate for the loss of high-frequency displacement information caused by a low GNSS sampling rate after the carrier attitude changes. At the same time, adopting the attitude correction method proposed in this paper can also effectively improve situations where the fusion effect deteriorates due to attitude changes. But, currently, there is still room for improvement in the algorithm: the calculation of the position and attitude of the four antennas uses a relatively simple least squares method and, in the future, the method of adjusting the GNSS network with multiple antennas can be used to improve the accuracy of positioning and attitude measurements. In addition, in extreme weather conditions, the effectiveness of network adjustment methods may be poor due to the deterioration of antenna results. Therefore, research can be conducted on GNSS ambiguity fixation and real-time solution strategies. Finally, the current attitude information is obtained through GNSS interpolation. In order to improve the accuracy and reliability of the attitude data, gyroscopes can be added and fused with the attitude data of accelerometers in the future.

## Figures and Tables

**Table 1:** The parameters of the GNSS receiver.

Parameters	Technical Indicators
Frequency point	BDS B1I/B2I1 GPS L1/L2 GLONASS L1/L2 Galileo E1/E5b QZSS L1/L2
Data format	NMEA-0183, Unicore
Differential data	RTCM 3.0/3.2/3.3
Data update rate	5 Hz
Single-point positioning (RMS)	Plane: 1.5 m; Elevation: 2.5 m
DGPS (RMS)	Plane: 0.4 m; Elevation: 0.8 m
RTK (RMS)	Plane: 1 cm + 1 ppm; Elevation: 1.5 cm + 1 ppm
Time accuracy (RMS)	20 ns
Directional accuracy (RMS)	0.2°/1 m baselien
Speed accuracy (RMS)	0.03 m/s

**Table 2:** The parameters of the accelerometer.

Range	±2	±4	±8	±10	±20	±40	Unit
Resolution interval value (@1Hz)	<1	<1	<1	<1	<1	<1	mg (max)
Nonlinearity	<0.5	<0.8	<1	<1	<1	<1	%FS (max)
Bandwidth (3DB)	500	500	500	500	500	500	Hz
Cross-axis sensitivity	1	1	1	2	2	2	%
Lateral vibration sensitivity ratio	1	1	2	5	5	5	%
Resonance frequency	2.4	2.4	2.4	5.5	5.5	5.5	Hz
68 protocol automatic output rate	5 Hz, 10 Hz, 25 HZ, 50 Hz, 100 Hz, 200 Hz, 500 Hz, 1000 Hz						
MODBUS automatic output rate	10 Hz, 25 Hz, 50 Hz						
Output interface	RS232/RS485/TTL						
Communication protocol	68 protocol and MODBUS RTU protocol						

## Data Availability

The data presented in this study are available on request from the corresponding author.
